# Mid-Infrared Dispersion Spectroscopy as a Tool for Monitoring Time-Resolved Chemical Reactions on the Examples of Enzyme Kinetics and Mutarotation of Sugars

**DOI:** 10.1177/00037028241258109

**Published:** 2024-07-25

**Authors:** Alicja Dabrowska, Andreas Schwaighofer, Bernhard Lendl

**Affiliations:** 1 Research Division of Environmental Analytics, Process Analytics and Sensors, Institute of Chemical Technologies and Analytics, 27259 Technische Universität Wien, Vienna, Austria; 2 Analytical Development Europe, R&D Pharmaceutical Science, Baxalta Innovations GmbH (part of Takeda), Vienna, Austria

**Keywords:** Mid-infrared spectroscopy, MIR, dispersion spectroscopy, quantum cascade laser, QCL, liquid-phase analysis, carbohydrates, enzyme activity, two-dimensional correlation spectroscopy, 2D-COS

## Abstract

Ongoing technological advancements in the field of mid-infrared (MIR) spectroscopy continuously yield novel sensing modalities, offering capabilities beyond traditional techniques like Fourier transform infrared spectroscopy (FT-IR). One such advancement is MIR dispersion spectroscopy, utilizing a tunable quantum cascade laser and Mach–Zehnder interferometer for liquid-phase analysis. Our study assesses the performance of a custom MIR dispersion spectrometer at its current development stage, benchmarks its performance against FT-IR, and validates its potential for time-resolved chemical reaction monitoring. Unlike conventional methods of IR spectroscopy measuring molecular absorptions using intensity attenuation, our method detects refractive index changes (phase shifts) down to a level of 6.1 × 10^–7^ refractive index units (RIU). This results in 1.5 times better sensitivity with a sevenfold increase in analytical path length, yielding heightened robustness for the analysis of liquids compared to FT-IR. As a case study, we monitor the catalytic activity of invertase with sucrose, observing the formation of resultant monosaccharides and their progression toward thermodynamic equilibrium. Anomalous refractive index spectra of reaction mixtures, with substrate concentrations ranging from 2.5 to 25 g/L, are recorded, and analyzed at various temperatures, yielding Michaelis–Menten kinetics findings comparable to the literature. Additionally, the first-time application of two-dimensional correlation spectroscopy on the recorded dynamic dispersion spectra correctly identifies the mutarotation of reaction products (glucose and fructose). The results demonstrate high precision and sensitivity in investigating complex time-dependent chemical reactions via broadband refractive index changes.

## Graphical abstract

**Graphical abstract:**
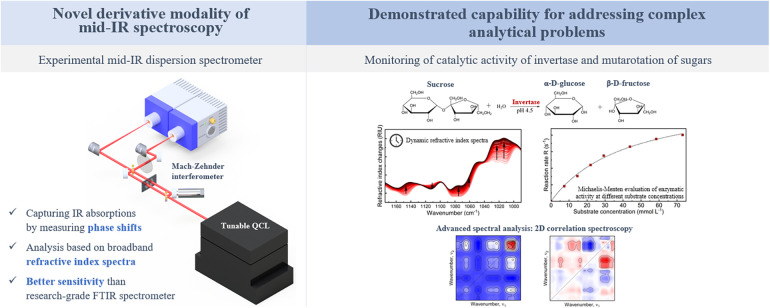
This is a visual representation of the abstract.

## Introduction

Mid-infrared (MIR) spectroscopy, headed by Fourier transform infrared spectroscopy (FT-IR), is a powerful analytical technique that is widely used in research, quality control, and various industrial applications for identifying chemical compounds, monitoring reactions, and studying molecular structures.^1,2^ Operating within the MIR regime (400–4000 cm^–1^) triggers fundamental vibrations of chemical bonds and functional groups in samples of diverse forms, enabling highly specific, non-destructive, and direct, label-free analysis.

Mid-infrared (MIR) dispersion spectroscopy is a powerful variant of MIR spectroscopy that expands the possibilities of conventional approaches such as FT-IR-based absorption spectroscopy. Instead of being reliant on intensity attenuation for detecting infrared (IR) absorptions, this technique detects alteration in the phase Δφ of the electromagnetic wave as it traverses the absorbing medium. The phase shifts arise from characteristic anomalous behavior in the real part of the refractive index function of the sample around IR absorption frequencies. Hence, the foundation of this technique lies in the fact that the refractive index function of the sample *ñ* describing the interplay of light with the respective medium, is a complex quantity, variable with frequency, and is represented as *ñ = n + ik*, wherein the imaginary part *k* is the index of absorption, describing the attenuation of light, and the real part *n* is the index of refraction, accounting for phase shifts of the travelling wave.^3,4^ These two optical properties, *n* and *k*, are interrelated facets of the same phenomenon, namely absorption, and alterations in one will lead to corresponding changes in the other. Hence, equivalent information about the examined sample can be derived by transducing either of these mentioned properties. Nevertheless, by providing access to the optical phase information, dispersion spectroscopy has the potential to overcome some of the well-known limitations associated with conventional intensity-based (index of absorption-based) methodologies. Unlike classic absorption spectroscopy, the technique is decoupled from the intensity noise of the employed source. The measured signal, namely, phase shift, develops only in the presence of the analyte, linearly increasing with both the thickness and concentration of the sample. This is in stark contrast to the logarithmic decay of intensity encountered in conventional absorption measurements, posing challenges to the sensitivity and dynamic range of the employed detectors.^5^ The proposed technique delivers background-free detection with higher and constant sensitivity, along with an extended dynamic range, even for optically thick (highly concentrated) samples, surpassing the quantitative capabilities of the Beer–Lambert law.^6–9^ Moreover, significantly different characteristics in signal-to-noise ratio (SNR) highlight the potential to extend the optimal pathlength for liquid phase analysis, leading to lower detection limits and greatly facilitating sample handling through reduced pressure drops in liquid transmission cells.^7,10^

Hence, dispersion sensing holds multiple advantages for liquid-phase analysis, and yet the sample analysis based on phase information in the MIR field is often overlooked and undervalued. Nowadays, the access to the phase information can be straightforwardly achieved by combining interferometry and the capabilities of coherent and intrinsically polarized IR sources, namely quantum cascade lasers (QCLs),^11^ which additionally offer broad tunability (>200 cm^–1^) and generate high-power IR beams (mW–W). In 2018, a proof-of-concept setup for dispersion spectroscopy of liquid-phase samples was introduced, utilizing a tunable QCL coupled to a Mach–Zehnder Interferometer (MZI).^12^ Successive works,^7,13–15^ not only refined the initial setup by enhancing acquisition speed, thermal and mechanical stability, and implementing a readout scheme that fully harnessed the aforementioned advantages, but also showcased its versatility by analyzing binary and complex mixtures of carbohydrates^13^ and proteins.^14^ Nowadays, after overcoming the method development phase, the setup is capable of addressing real chemical challenges.

Invertase, also known as β-fructofuranosidase, is a valuable enzyme in both biological and commercial contexts.^16^ It is a vital glycoprotein responsible for catalyzing the hydrolysis of sucrose into its constituent sugars, glucose and fructose, a mixture known as invert sugar. Consequently, invertase plays an important role in carbohydrate metabolism in various organisms, including plants, yeast, honeybees, and other microorganisms, regulating their biological functions related to energy production and growth. Furthermore, this enzyme finds widespread applications in various industrial sectors, particularly in food industry, where it is effectively used on a large-scale in production of invert sugar syrup, a soluble, sweetener commonly added to foods, beverages, and confectionery products.^17^

Given its broad-ranging function and application, invertase has been well studied in the literature, including the use of techniques such as FT-IR.^18,19^ This makes it an excellent model candidate to benchmark our novel approach. In this study, we will assess whether complex dynamic chemical processes, such as enzyme kinetics, can be investigated solely relying on the broadband refractive index information offered by the new modality and the latest EC-QCL–MZI setup. This will be complemented by a powerful analytical tool such as two-dimensional correlation spectroscopy (2D-COS),^20–23^ a method that has yet not been applied to a broadband MIR refractive index data set.

## Materials and Methods

### Experimental Setup for MIR Dispersion Spectroscopy

**Figure 1.**
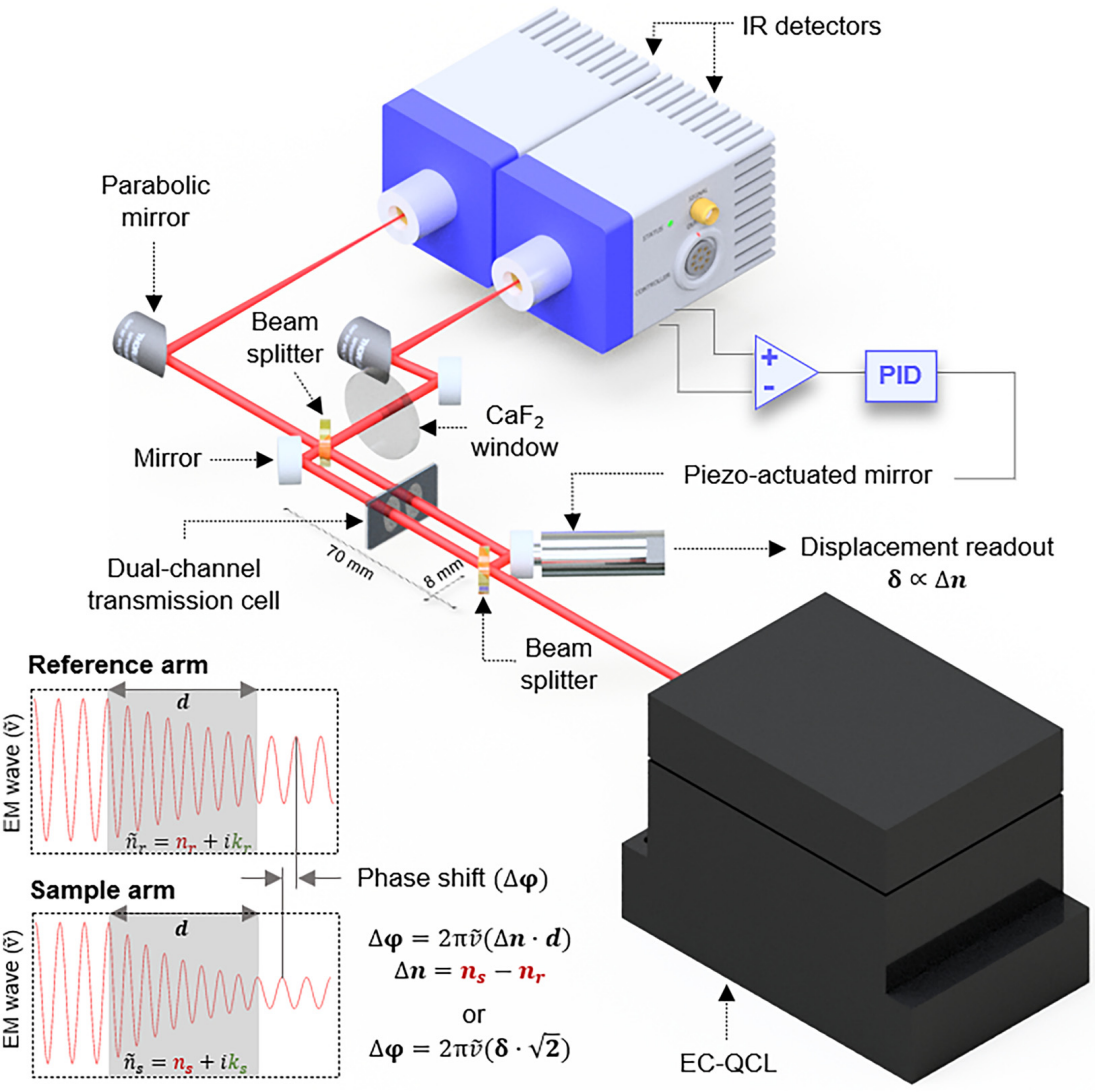
Experimental setup for MIR dispersion spectroscopy of liquids based on a tunable QCL and a compact free-space Mach–Zehnder interferometer.

A comprehensive description of the fundamental principle of the MIR dispersion spectrometer can be found in our previous study.^7^ The experimental setup, as employed in this study, is illustrated in Figure 1. It consists of a compact free-space Mach–Zehnder interferometer (70 × 8 mm) illuminated by a latest generation thermoelectrically (TE) cooled EC-QCL (Hedgehog, DRS Daylight Solutions Inc.) tunable from 935 to 1175 cm^−1^. The laser is operated in pulsed mode with a pulse repetition rate of 1.5 MHz, a duty cycle of 30%, and a current of 1400 mA. These parameters were selected to maximize the average output power (∼110 mW). Upon splitting at the first ZnSe 50 : 50 beamsplitter (Thorlabs BSW705), the two beams traverse a custom-made temperature-controlled transmission cell simultaneously filled with a reference (solvent) and a sample solution (solvent + analyte) for analysis. The cell is made of a pair of calcium fluoride (CaF_2_) windows (25 × 15 mm, 1 mm thickness, wedged 10 arcmin; Crystran Ltd.) separated by a polytetrafluoroethylene (PTFE) spacer.^7,15^ The transmission pathlength was optimized to 170 µm, ensuring the highest SNR in phase-shift (dispersion) measurements within the investigated spectral range for the aqueous sample matrix.^7^ Following recombination at the second identical beamsplitter, the resulting interfering beams (outputs) are focused onto two closely matched TE cooled mercury cadmium telluride (MCT) detectors (PVI-4TE-10.6, Vigo Systems S.A.; detectivity ≥2.0 × 10^9^ cm Hz^1/2^/W at 10.6 µm, bandwidth: DC-15 MHz). Although both outputs contain identical information about the sample, they exhibit opposite behaviors: while one detector records constructive interference, the other detects destructive interference. This is used to conveniently identify the quadrature condition of the MZI (the point of the highest sensitivity in the system), representing the midpoint between constructive and destructive conditions, while also exploiting the advantages of the balanced detection scheme. Consequently, the detector signals are subtracted using a differential input of a lock-in amplifier (MFLI, Zurich Instruments AG). This differential signal (set point 0) serves as the input for a proportional–integral–derivative servo closed loop in a quadrature point locking scheme. This loop controls the movement of a piezo-actuator (P-841.1, Physik Instrumente; 0.3 nm resolution, 15 µm travel range; 2 ms settling time) attached to one of the interferometric mirrors, actively counterbalancing for relative phase shifts generated between the reference and sample arm throughout the laser's spectral scanning. The piezo-mirror movement is tracked by an internal strain gauge position sensor, and the measured displacement is proportional to the refractive index (dispersion) spectrum of the sample. To achieve optimal common-mode noise cancellation, a CaF_2_ window is employed at one output of the MZI. It optically compensates for a factual unequal splitting ratio of the beamsplitters. It also ensures that the fringe contrast remains consistent across both outputs, and aids in correctly identifying the quadrature point. The laser sweep rate is set to 80 cm^–1^ s^–1^, enabling the piezo-actuator to accurately represent the signal (Figure S1, Supplemental Material). The opto-mechanical part of the experimental spectrometer is housed within a compact 22 × 30 cm enclosure. To maintain stable internal temperature, a water-cooled breadboard serves as the instrument's base, in combination with a liquid cooling system (ThermoCube Liquid-to-Liquid Solid State Cooling Systems). Furthermore, the compartment is gently purged with dry air at 5 L/min to achieve 0% humidity, reducing the impact of this parameter on the fluctuations of the refractive index of the air.

### Spectra Acquisition and Post-Processing

The IR dispersion spectrum is obtained by recording the changing piezo-displacement signal as the laser scans across the available spectral range. The signal is directly accessed via a built-in data acquisition module within the lock-in amplifier using a Python API. In addition, a final spectrum is calculated as Δ*n*(ṽ) = δ $\sqrt{2}\;/d$ , where δ $\sqrt{2}$ represents the piezo-displacement corrected for diagonal movement of the mirror, and *d* represents the applied transmission pathlength. To account for various deviations such as the phase-shift dependence on wavelength, contributions from the solvent matrix, dispersion of transmissive optics, and alignment imperfections within the setup, an initial calibration spectrum is acquired by filling both the reference and sample channels of the transmission cell with a solvent solution. This spectrum is subsequently subtracted from all the later recorded sample spectra. The number of scans averaged per spectrum can be adjusted based on the objectives of the study and the desired noise level. For the setup characterization and benchmarking, 100 scans are averaged per spectrum, resulting in a total acquisition time of 300 s. On the other hand, for dynamic reaction monitoring, a single scan is used per spectrum, corresponding to an acquisition time of 3 s. Before further evaluation, the spectra are filtered using a Savitzky–Golay filter with an order of 3 and a window size of 85 points, corresponding to a spectral resolution of 2 cm^–1^.

### Fourier Transform Infrared Spectroscopy (FT-IR)

The reference FT-IR absorption spectra were measured on a Vertex 80v spectrometer (Bruker Corporation) equipped with a Globar and a liquid-nitrogen-cooled MCT detector (D* = 4.0 × 10^10^ cm Hz^1/2^/W at 9.2 µm) and a Tensor 37 FT-IR spectrometer equipped with a deuterated L-alanine doped triglycine sulfate (DLaTGS) detector (D* = 6.0 × 10^8^ cm Hz^1/2^/W at 9.2 µm). Samples were measured at 25 °C in a transmission cell consisting of two 2 mm thick CaF_2_ windows and a 25 µm PTFE spacer. A total of 1416 (Vertex 80v, scanner speed 80 kHz) and 382 (Tensor 37, scanner speed 20 kHz) scans were averaged per spectrum, leading to an acquisition time of ∼300 s. Spectra were recorded with a resolution of 2 cm^−1^ in double-sided acquisition mode and were calculated using a Blackman–Harris three-term apodization function and zero filling factor of 2. The interferometer compartment of the Vertex 80v FT-IR spectrometer was evacuated (2.88 hPa). Spectra were evaluated with the OPUS 8.1 software package (Bruker Corporation).

### Reagents and Samples

Sucrose (≥99.5%), invertase from baker's yeast (*Saccharomyces cerevisiae*, Grade VII, ≥334 U/mg), and sodium acetate trihydrate (≥99.5%) were obtained from Sigma-Aldrich. The substrate stock solution (100 g/L) and enzyme stock solution (20 U/mL) were prepared in 0.05 mol/L acetate buffer (pH 4.5) and stored at 4 °C for <3 weeks. For the mutarotation study, appropriate amounts of anhydrous α-D-glucose (96%; Sigma-Aldrich) and D-(–)-fructose (≥99%) were dissolved in the buffer directly prior to the measurements. D-(+)-glucose (≥99.5%; Sigma-Aldrich) was diluted in distilled water to appropriate concentrations and after equilibration was used for the assessment of the setup's performance.

## Results and Discussion

### Evaluation of Setup Performance and Comparison to FT-IR

The performance of the laser-based IR dispersion spectrometer was evaluated and benchmarked against conventional FT-IR. In this context, IR dispersion and absorption spectra of glucose in aqueous solutions were recorded with the respective modality (Figure S2, Supplemental Material) and used to derive performance parameters for each instrument, such as, limit of detection (LOD), and limit of quantification (LOQ). The overall results of the comparison are summarized in Table I. For each modality, the most optimal analytical pathlength that yielded the highest SNR was selected. Similar acquisition times were chosen for comparison.

**Table I.** Comparison of performance parameters between EC-QCL–MZI dispersion spectroscopy and FT-IR. The LOD and LOQ were defined as 3× and 10 × N/S, respectively, where N is the noise level and S is the signal corresponding to the slope of the calibration curve.

	Measurement time (s)/scans	Path length (µm)	Noise level	SNR	LOD (g/L)	LOQ (g/L)	Dynamic range	Spectral range (cm^–1^)
Dispersion spectroscopy (EC-QCL–MZI)	300/100	170	6.10 × 10^–7^ (RIU)	409	0.007	0.024	0.007–40 g/L (5700)	935–1175
Routine FT-IR (Tensor 37)	300/382	25	3.01 × 10^–4^ (AU)	147	0.162	0.542	0.162–180 g/L* (1100)	400–4000
High-end FT-IR (Vertex 80v)	300/1416	25	2.00 × 10^–5^ (AU)	276	0.011	0.052	0.011–180 g/L* (16 000)	400–4000

*The upper threshold was determined as the sample concentration that leads to an absorbance of 1 absorbance unit (AU).

The results of the performance comparison show that the achieved LOD of 7 mg/L (0.7 mg/dL) for glucose detection by dispersion spectroscopy is >20 times better than that of a routine FT-IR spectrometer equipped with a DLaTGS detector operating at room temperature. This significant improvement can be attributed not only to the approximately sevenfold increase in analytical pathlength, enabled by significantly higher spectral power density of the laser source (>10^4^ or more) and more sensitive detector but also the utilization of the dispersion mode, which not only allows to maximize the applicable pathlength, due to its linear SNR behavior, but also provides greater resilience of the balanced phase shift detection against environmental noise and intensity fluctuations.^7^ Both techniques probe comparable amounts of material despite utilizing different pathlengths, with ∼0.5 µL for dispersion spectroscopy and 0.7 µL for FT-IR. This is attributed to the approximately threefold smaller diameter of the laser beam at the sample position. Furthermore, the dispersion spectrometer also outperforms the high-end FT-IR instrument equipped with 20 times more sensitive LN_2_-cooled MCT detector. It achieves a LOD that is ∼1.5× lower than that of a research-grade spectrometer, showcasing the outstanding performance of the custom-made instrument and ensuring its capability for high-quality measurements without the necessity for LN_2_ detector cooling. During the course of instrumental developments, this also marks the first instance where this alternative modality has shown competitive sensitivity over a research-grade FT-IR instrument for liquid analysis. It is worth noting, that the reported noise levels has improved by approximately fivefold for the same spectral range and a comparable acquisition time, when compared to our previous study.^7^ This is largely due to: (i) incorporation of the latest generation laser source with comparably higher power output (13× higher), improved scan-to-scan alignment and wavelength reproducibility, (ii) simplification of the readout scheme through the utilization of internal DAQ module of the lock-in amplifier instead of relying on external DAQ cards, and (iii) improved sensitivity through compensation for unequal splitting ratio of the beamsplitters. However, the broader spectral coverage of the FT-IR spectrometers provides greater versatility, while the reduced dynamic range of the custom-made instrument can be attributed to a significantly increased pathlength that essentially limits the light throughput.

**Figure 2.**
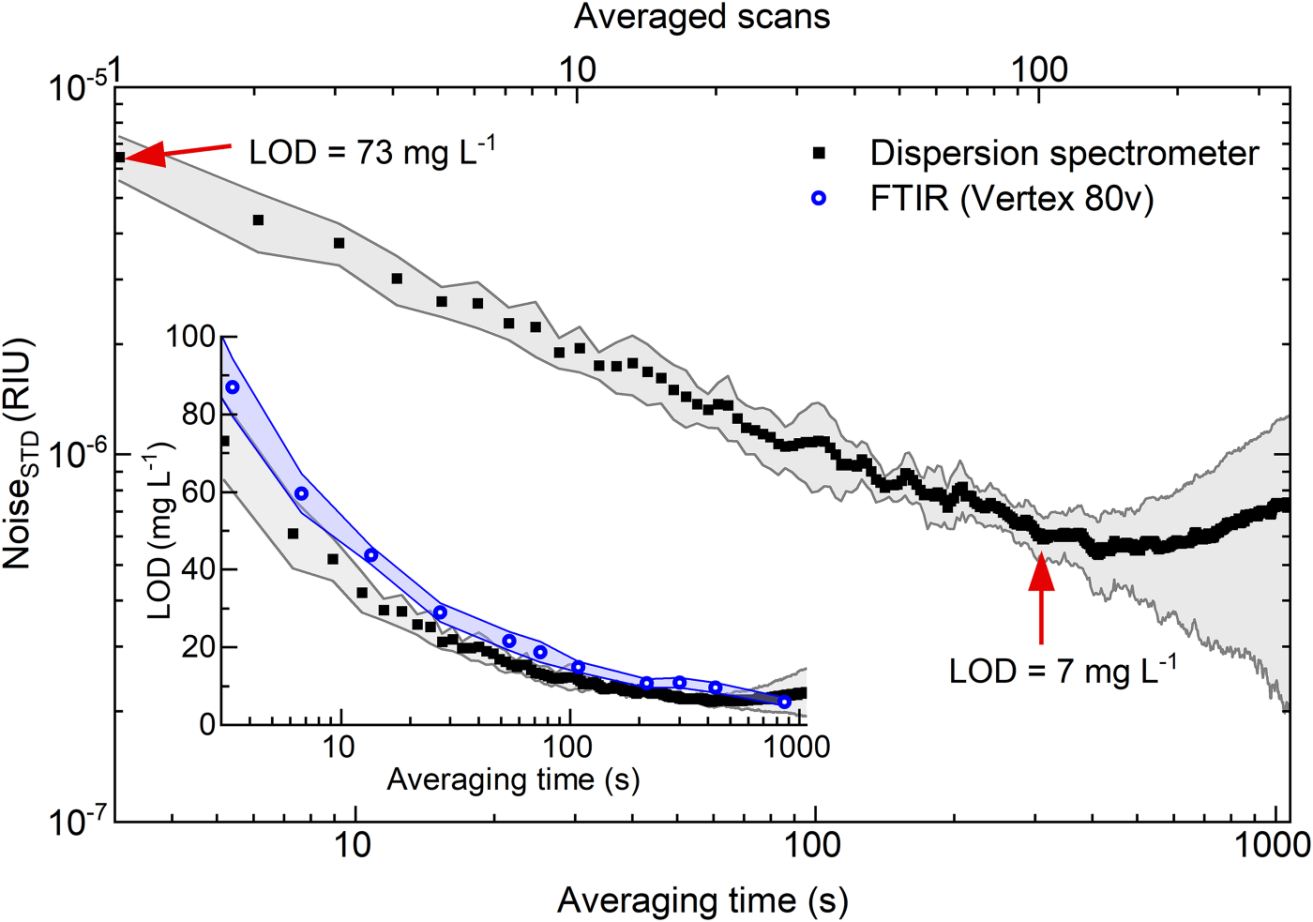
The noise level of dispersion spectra as a function of integration time and number of scans used for averaging. Gray/blue shades represent standard deviations of the mean values (average of 10 measurements). Inset: The resulting limits of detection for the dispersion spectrometer and the Vertex 80v FT-IR spectrometer.

An additional analysis was conducted to characterize the temporal noise behavior in the experimental spectrometer, as illustrated in Figure 2. The noise levels were assessed by calculating the standard deviation of background dispersion spectra (water vs. water), measured at various integration times within the spectral range of 1000 to 1175 cm^–1^. Signal averaging improves the SNR by decreasing the noise level through averaging of high-frequency noise components within the spectra. In the initial 300 s of averaging time (100 scans), an ∼9.5-fold reduction in high-frequency noise can be achieved, resulting in a noise level of 6.1 × 10^–7^/refractive index units (RIU). After that, the contribution from low-frequency noise (temperature effect, vibrational noise, and random walk) starts to affect the system. For rapid monitoring of time-dependent reactions, as later presented in this study, only one scan is used, where the noise remains at an acceptable level of 6.46 × 10^–6^/RIU. The inset further illustrates the resultant limits of detection at different acquisition times for both the dispersion spectrometer as well as the Vertex 80v spectrometer, confirming higher sensitivity of the custom-made instrument for the integration times <600 s. However, beyond this threshold, the FT-IR spectrometer begins to surpass our instrument's performance owing to its robust design, and vacuum environment within the interferometer compartment.

### Monitoring of an Enzymatic Reaction

The dispersion spectrometer was employed to monitor enzymatic activity of invertase. Invertase catalyzes the irreversible hydrolysis of the glycosidic linkage present in sucrose, leading to production of equal parts of β-fructose and α-glucose (invert syrup). This chemical reaction can be directly monitored using IR spectroscopy, because the reaction substrate and the reaction products show characteristic IR absorption signatures in the investigated spectral region of 990–1175 cm^–1^, attributed mainly to C–O and C–C stretching modes as well as C–O–H bending vibrations of the saccharides (Figure S3, Supplemental Material).^24^ The rapid negative change of refractive index with increasing wavenumber (d*n*/dṽ < 0), as observed in the dispersion spectra, aligns with the peaks of the absorption bands in the reference absorption spectra. These distinctive regions, characterized by anomalous behavior, are typically used for qualitative and quantitative analysis in dispersion spectroscopy.

**Figure 3.**
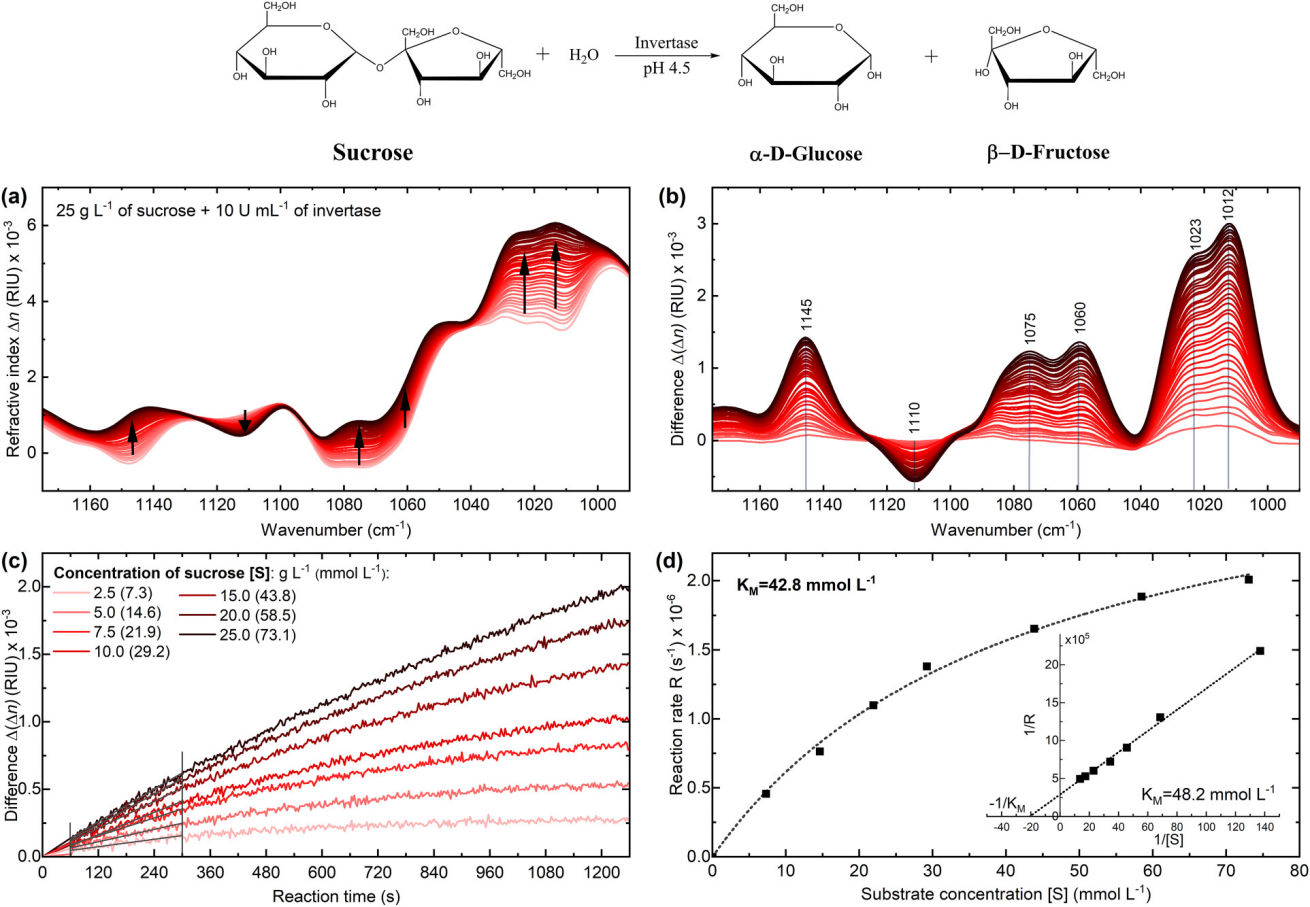
(a) Infrared (IR) dispersion spectra of the enzymatic reaction catalyzed by invertase at 25 °C and pH 4.5 monitored throughout 20 min of the reaction time. A single spectrum is plotted every 30 s. (b) Difference spectra of the reaction calculated by subtracting the spectrum of the substrate solution without the enzyme from the spectra of the reaction mixture. (c) Progression curves of the enzymatic reaction of 5 U/mL invertase with seven different substrate concentrations evaluated at 1012 cm^–1^. (d) Reaction rate versus substrate concentration for determination of the Michaelis–Menten constant (inset: Lineweaver–Burke plot).

The enzymatic activity was tracked by recording dispersion spectra of the reaction mixture, consisting of sucrose and invertase, over a course of 20 min, at a temperature of 25 °C, and a pH of 4.5. Figures 3a and 3b show the acquired spectra, including the difference spectra obtained by subtracting a spectrum of the substrate without the enzyme. Within the observation window, the consumption of substrate and the subsequent formation of reaction products resulted in several changes in refractive index across the examined spectral range, with the most notable change observed at 1012 cm^–1^. The reaction was repeated for different initial substrate concentrations ranging from 2.5 to 25 g/L (7.3–73.1 mmol/L) and the mentioned increase at 1012 cm^–1^ was used for further evaluation. In Figure 3c, the difference band heights of each of the reactions were plotted versus the reaction time. Subsequently, the initial slopes of the progression curves were determined by a linear fit between 60 and 300 s of the reaction time. This was the time range when a linear relationship between substrate surplus and reaction time was ensured. The first 60 s was the time required for sample injection and start of acquisition after the proper amount of enzyme was added to the substrate. The obtained initial reaction rates were plotted versus the substrate concentration and showed a good fit (*R*^2 ^> 0.996) to the Michaelis–Menten equation (Figure 3d), which is typically used to describe enzyme reactions under conditions of zero-order kinetics at the beginning of the reaction. Based on this evaluation, a Michaelis–Menten constant (*K*_M_) of 42.8 mmol/L was determined. To further validate this finding, a Lineweaver–Burke plot was constructed on the data, also yielding a *K*_M_ value of 48.2 mmol/L. Both values are in good agreement with previously reported literature values of ∼44 mmol/L for invertase.^18,25^

### Temperature Dependence on the Enzyme's Activity

**Figure 4.**
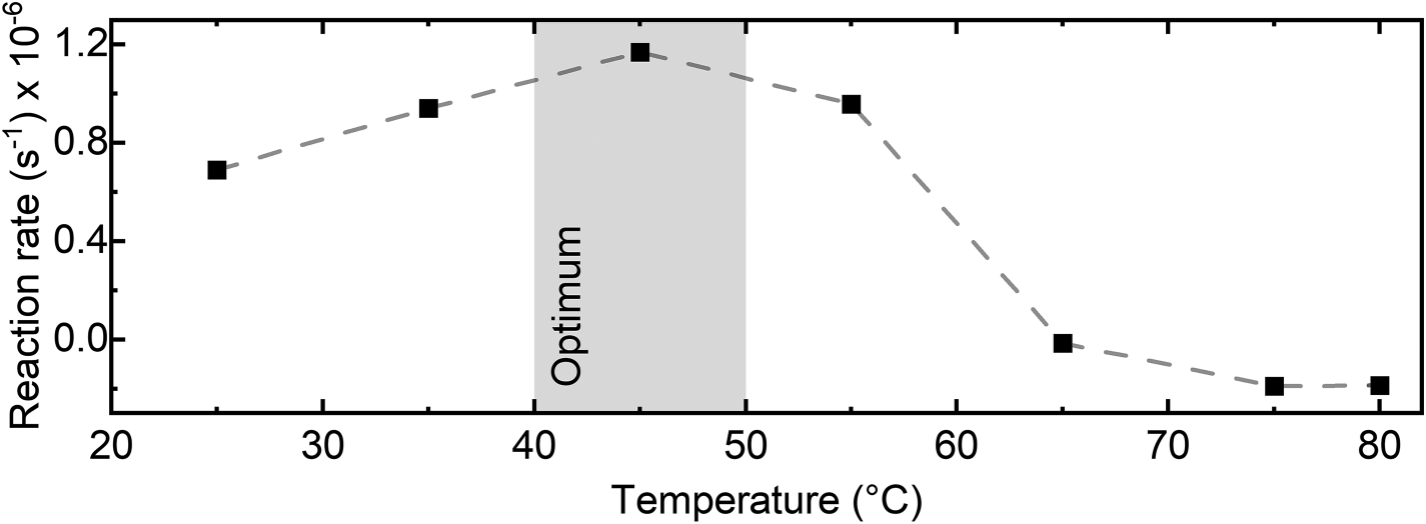
Enzymatic activity of invertase measured at various temperature conditions (pH 4.5). The reaction rates were evaluated between 60 and 300 s of the reaction time.

Assessment of the enzyme's optimum temperature provides valuable information, facilitating better understanding, control, and optimization of bioprocesses involving the enzyme. Firstly, long-term stability of the noise level in the dispersion spectrometer was evaluated under various temperature conditions using a similar approach as described in the Evaluation of Setup Performance and Comparison to FT-IR section above. The assessment was conducted in the range between 25 and 80 °C by adjusting the temperature of the transmission cell. The results are displayed in Figure S4 (Supplemental Material). Although the noise level is best maintained in the range between 25 and 65 °C, stable measurements can still be performed up to 80 °C. Hence, the dispersion spectra of the reaction mixture consisting of 10 g/L of sucrose and 2.5 U/mL of invertase were repetitively measured at a 10 °C interval. The difference spectra were calculated and assessed as before. Figure 4 shows the changes in the rate of the enzymatic reaction as a function of temperature, highlighting the optimum temperature range for maximum catalytic efficiency and stability of invertase between 40 and 50 °C. At temperatures exceeding 60 °C, the enzyme experiences significant inhibition due to irreversible structural and functional changes of the protein molecule through denaturation. These findings agree well with literature.^25^

### Two-Dimensional Correlation Spectroscopy (2D-COS) Analysis of the Enzymatic Reaction

Normalization of difference spectra presented in Figure 3b exposed notable inconsistency and variability across the investigated spectral range, which should not be observed for a single consistent reaction rate (Figure S5, Supplemental Material). The biggest changes occur in the region between 1027 and 1087 cm^–1^ with a peak centered at 1060 cm^–1^. This result indicates the presence of an additional reaction (component) taking place concurrently to the sucrose breakdown. According to understanding of the reaction, this must be related to the mutarotation of reaction products, glucose and fructose. To confirm this hypothesis, a 2D-COS analysis of the invertase reaction spectra was conducted to provide a more precise mapping of the asynchronous regions. Simultaneously, a detailed study on the mutarotation of glucose and fructose was conducted to determine whether the observed changes align with those associated with sugar mutarotation (for these results and a comprehensive explanation of the mutarotation phenomenon, please refer to the subsequent section, Mutarotation of the Reaction Products.

**Figure 5.**
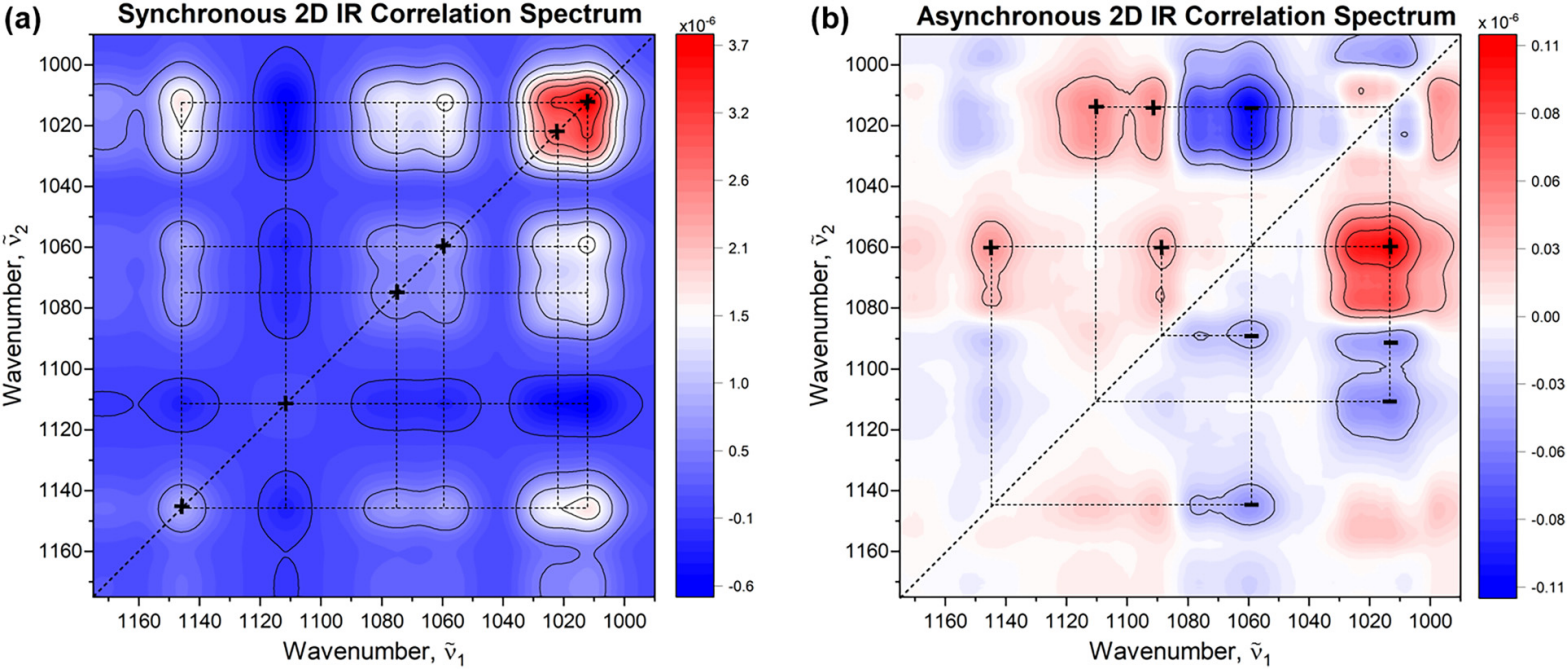
(a) Synchronous and (b) asynchronous 2D IR correlation maps obtained from time-dependent (dynamic) dispersion spectra of sucrose and invertase. The first spectrum, representing the pure substrate, was used as the reference. The Matlab-based mat2Dcorr toolbox under CC-BY-NC-SA 4.0 license was used for performing the 2D-COS analysis.^26^

Using 2D-COS elucidates subtle changes, cross-correlations, and interaction between different vibrational modes or functional groups, providing valuable insights into dynamic changes occurring in the sample within the well-defined observation period. By spreading the spectral variations over the second dimension, 2D-COS analysis is able to determine spectral and temporal relationships within the data, by using a set of rules associated with the signs of correlation peaks (i.e., Noda's rules).^20,21^ The intensities of the 2D-COS map represent the quantitative measure of a comparative similarity or dissimilarity of spectral intensity variations measured at two different spectral variables ṽ_1_ and ṽ_2_.^20,21^ In this context, the dynamic dispersion spectra of 25 g/L of sucrose and 10 U/mL of invertase (25 °C, pH 4.5) were subjected to 2D-COS analysis. The resulting synchronous and asynchronous 2D-COS maps of the reaction under investigation are depicted in Figure 5. The synchronous map shows auto peaks at 1012, 1023, 1060, 1075, 1010, and 1145 cm^–1^ at which simultaneous spectral changes occur, with the most pronounced peak at 1012 cm^–1^, which agrees well with previously shown 1D difference spectra. It also marks the most susceptibility of the corresponding spectral regions to external perturbation. Positive cross-peaks at off-diagonal positions, i.e., (1012, 1060), (1012, 1075), (1012, 1145), (1060, 1145), (1075, 1145), and (1060, 1075) indicate the regions that exhibit the same pattern of intensity changes. In turn, negative cross-peaks at (1012–1075, 1110) and (1110, 1145) suggest the opposite direction of spectral intensity changes and already highlight the presence of another component. As for the asynchronous map, which develops only in the presence of uncoordinated (out-of-phase) variations in the IR signal, the positive and negative peaks centered at (1012, 1060) and (1012, 1110), respectively, suggest that the changes at ṽ_2_ occur after the changes at ṽ_1_ associated with decreasing concentration of sucrose (ṽ_1_→ṽ_2_). The negative peak is interpreted inversely due to the negative sign in the synchronous plot at the corresponding coordinate. On the other hand, the negative peaks at (1060, 1089), (1060, 1145), and (1012, 1092) indicate that the changes at ṽ_2_ occur before or at higher rate than the changes at ṽ_1_(ṽ_1_←ṽ_2_). To verify the origin of the identified peaks, a subsequent investigation focused on the mutarotation of glucose and fructose was conducted.

### Mutarotation of the Reaction Products

Mutarotation, also known as anomerization or change in rotation, refers to the spontaneous interconversion between the α- and β-anomers of a carbohydrate upon dissolution in water. This process continues through the opening and closing of the hemiacetal or hemiketal ring structure until an equilibrium between these two forms is established. It is thus an intrinsic property of reducing sugars, which encompasses sugars with a free aldehyde (R–CH=O) or ketone (R–C=O–R′) group, such as glucose and fructose produced in the enzymatic hydrolysis of sucrose catalyzed by invertase.^27^ Mutarotation is commonly investigated through techniques such as polarimetry, nuclear magnetic resonance spectroscopy, or high-performance liquid chromatography.^28,29^ FT-IR has also demonstrated application in this context, offering a rapid and sensitive method for distinguishing between isomeric forms in aqueous solutions.^30,31^ However, recent advancements in laser tuning rates in conjunction with innovative low-noise techniques have made it increasingly more feasible to study mutarotation using laser-based variants of MIR spectroscopy.

**Figure 6.**
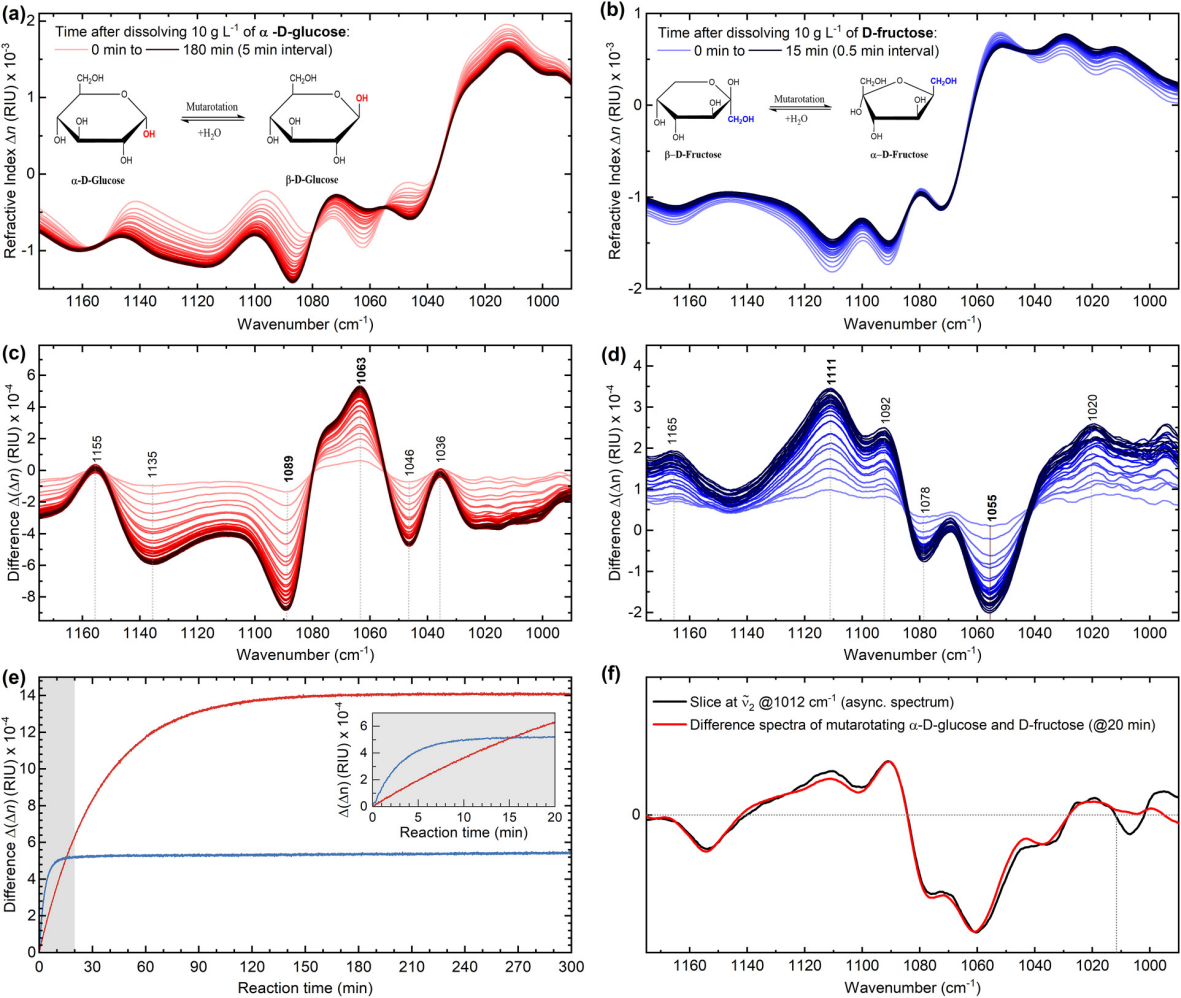
(a, b) Infrared (IR) dispersion spectra of α-D-glucose and D-fructose undergoing mutarotation (*T* = 25 °C, pH 4.5). (c, d) Difference spectra of the mutarotation. (e) Progression curves of the mutarotation of 10 g/L α-D-glucose (red) and 10 g/L D-fructose (blue) recorded via MIR dispersion spectroscopy. The amplitudes of dispersion change were evaluated between the local extrema observed at 1063 and 1089 cm^–1^ for D-glucose, and at 1055 and 1111 cm^–1^ for D-fructose. The inset shows the first 20 min of the mutarotation. (f) Qualitative comparison of the difference spectra measured for the mutarotation of α-D-glucose and D-fructose at the 20 min time stamp (red) with the asynchronous correlation slice of the enzymatic reaction at ṽ_2_ = 1012 cm^–1^.

In this context, Figure 6 shows the IR refractive index spectra measured via the QCL-IR dispersion spectrometer during the mutarotation process of α-D-glucose and D-fructose shortly after (<60 s) their dissolution in buffered water (pH 4.5). A single spectrum was recorded every 3 s and the reaction was continuously monitored for up to 5 h. Both monosaccharides exhibit notable changes in refractive index within the investigated spectral range associated with the rotation of groups around the anomeric carbon. Furthermore, Figure 6e shows the progression curves of the two reactions following first-order reaction kinetics.

First, the results demonstrate that anomerization process of α-D-glucose, which is known to undergo simple mutarotation between α- and β-forms through an open-chain *aldehydo* structure,^29^ lasts ∼3 h until fully (>99%) completed. In contrast, complex mutarotation of D-fructose seems to reach equilibrium in about 15 min after dissolution.^29^ It is important to note that the crystalline form of D-fructose, used for demonstration in this study, is primarily composed of β-D-fructopyranose and undergoes mutarotation after dissolution to at least five different tautomeric forms: α- and β-pyranose, α- and β-furanose, and the open chain *keto* form.^28^ The interconversion between each of those forms occurs at different rates and the conversion observed within the first minutes after dissolution can be accounted to β-pyranose to furanose forms transformation.^32^ Furthermore, a consistent increase in the Δn signal beyond the initial 15 min throughout the entire observation window suggests that the equilibrium has not been reached. The changes may potentially be linked to considerably slower pyranose-to-pyranose transformation.^28^

Secondly, the combination of the difference spectra measured for the two mutarotation cases, when compared with the slice of the previously illustrated asynchronous spectrum of the enzymatic reaction at v_2_ 1012 cm^–1^, yields highly overlapping results. This substantial spectral overlap, as depicted in Figure 6f, ultimately confirms that the 2D-COS analysis has indeed detected a secondary reaction involving mutarotation of the reaction products. Further validation was obtained by calculating 2D-COS maps from spectra of pure reaction components (refer to Figure S6, Supplemental Material), assuming no mutarotation of the products, and then comparing them to the maps shown for the enzymatic reaction. It underscores the high quality of the acquired data by our experimental spectrometer and sets a foundation for future investigations of similarly complex analytical problems.

## Conclusion

In conclusion, we introduced a new modality to monitor time dependent chemical reactions that is based on the analysis of refractive index changes caused by MIR absorptions. We hereby showcased that the dispersion spectroscopy not only compares favorably with but also surpasses, for the first time, the sensitivity of research-grade FT-IR under specified evaluation parameters, without the need for LN_2_ cooling. The process to achieve this advancement took time, but it mirrors the similar course of developments seen in laser-based absorption spectroscopy.^33–35^ Our validation study indicates that laser-based IR dispersion spectroscopy offers an alternative approach to absorption spectroscopy for elucidating structural and compositional changes in dynamic chemical systems within their native environments. Nevertheless, our approach provides a greater perspective toward more sensitive and robust liquid sensing. The technique was hereby successfully used to study enzyme kinetics and mutarotation in a label-free, and non-destructive manner. Importantly, this was achieved at significantly extended pathlength, with direct proportionality of the measured signal on the analyte's concentration, and inherent resilience towards intensity fluctuations. Moreover, the analysis of dispersion spectra with potent tools such as 2D-COS showcased that even the most subtle changes in the refractive index of the sample can reveal important information on reaction intermediates and hence on further, simultaneously occurring processes. This highlights the high quality of the obtained signal. Nonetheless, further actions to improve the system's noise floor will be taken in the future. These will involve a more monolithic design of the interferometer and the separation of the instrument's response time from the speed of the piezo-element by implementing an optical readout scheme. Finally, it is worth mentioning that there has been a notable trend in developing chip-scale MIR interferometric sensors transducing refractive index for rapid, compact, and on-line liquid analyses across diverse applications. In this context, the proposed system can serve as an excellent benchmark for the developed miniaturized devices.

## Supplemental Material

sj-docx-1-asp-10.1177_00037028241258109 - Supplemental material for Mid-Infrared Dispersion Spectroscopy as a Tool for Monitoring Time-Resolved Chemical Reactions on the Examples of Enzyme Kinetics and Mutarotation of Sugars Supplemental material, sj-docx-1-asp-10.1177_00037028241258109 for Mid-Infrared Dispersion Spectroscopy as a Tool for Monitoring Time-Resolved Chemical Reactions on the Examples of Enzyme Kinetics and Mutarotation of Sugars by Alicja Dabrowska, Andreas Schwaighofer and Bernhard Lendl in Applied Spectroscopy
