# Supplementary material for: Mid-Infrared Dispersion Spectroscopy as a Tool for Monitoring Time-Resolved Chemical Reactions on the Examples of Enzyme Kinetics and Mutarotation of Sugars
Source: Appl Spectrosc. 2024 Jul 25;78(9):982–92. doi: 10.1177/00037028241258109 (PMC11462787; doi:10.1177/00037028241258109)
Supplement: sj-docx-1-asp-10.1177_00037028241258109 - Supplemental material for Mid-Infrared Dispersion Spectroscopy as a Tool for Monitoring Time-Resolved Chemical Reactions on the Examples of Enzyme Kinetics and Mutarotation of Sugars [file sj-docx-1-asp-10.1177_00037028241258109.docx]

Supplemental material

**Mid-infrared dispersion spectroscopy as a tool for monitoring**

**time-resolved chemical reactions on the example of enzyme kinetics and mutarotation of sugars**

Alicja Dabrowska^1^, Andreas Schwaighofer^1,‡^, and Bernhard Lendl^1,^*

^1^ Research Division of Environmental Analytics, Process Analytics and Sensors, Institute of Chemical Technologies and Analytics, Technische Universität Wien, Getreidemarkt 9/164-UPA, 1060 Vienna, Austria

‡ Current address: Analytical Development Europe, R&D Pharmaceutical Science, Baxalta Innovations GmbH (part of Takeda), Industriestraße 131, 1220 Vienna, Austria

**Corresponding Author(s):**

* E-Mail: [bernhard.lendl@tuwien.ac.at](mailto:bernhard.lendl@tuwien.ac.at)

Postal address: Institute of Chemical Technologies and Analytics, Technische Universität Wien, Getreidemarkt 9/164-UPA, 1060 Vienna, Austria

**DESCRIPTION OF CONTENTS:**

Figure S1. The relationship between laser sweep rate and piezo-displacement signal.

Figure S2. Mid-IR dispersion and absorption spectra of glucose in aqueous solution recorded in transmission with the proposed system and an FTIR spectrometer, respectively.

Figure S3. Reference absorption spectra of sucrose, glucose, and fructose recorded by Vertex 80v in transmission compared to dispersion spectra recorded by laser-based dispersion setup.

Figure S4. Impact of the liquid transmission cell’s temperature on the noise level of the dispersion spectrometer.

Figure S5. Comparison of the normalized difference spectra of the enzymatic reaction catalyzed by invertase at selected reaction time stamps.

Figure S6. Synchronous and asynchronous 2D IR correlation maps calculated from spectra of pure reaction components, assuming no mutarotation of the reaction products.


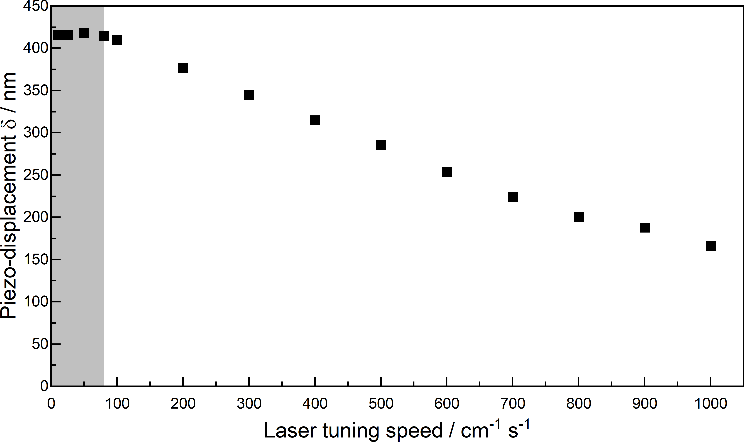


**Figure S1.** The relationship between laser sweep rate and piezo-displacement signal evaluated at 1012 cm^-1^, which corresponds to the local maximum of the dispersion spectrum of a 15 g L^-1^ glucose solution in aqueous medium. Using laser sweep rates > 80 cm^-^ ^1^ s^-1^ results in an imprecise representation of the analyte's dispersion spectrum and diminished sensitivity. Gray shaded area indicates the optimum sweep rate range.


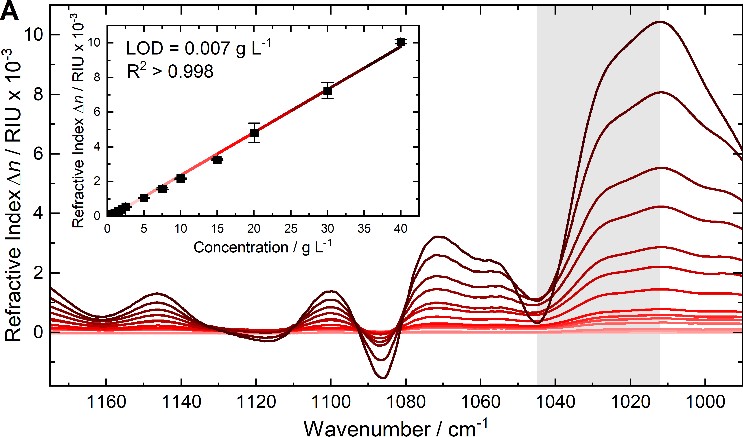


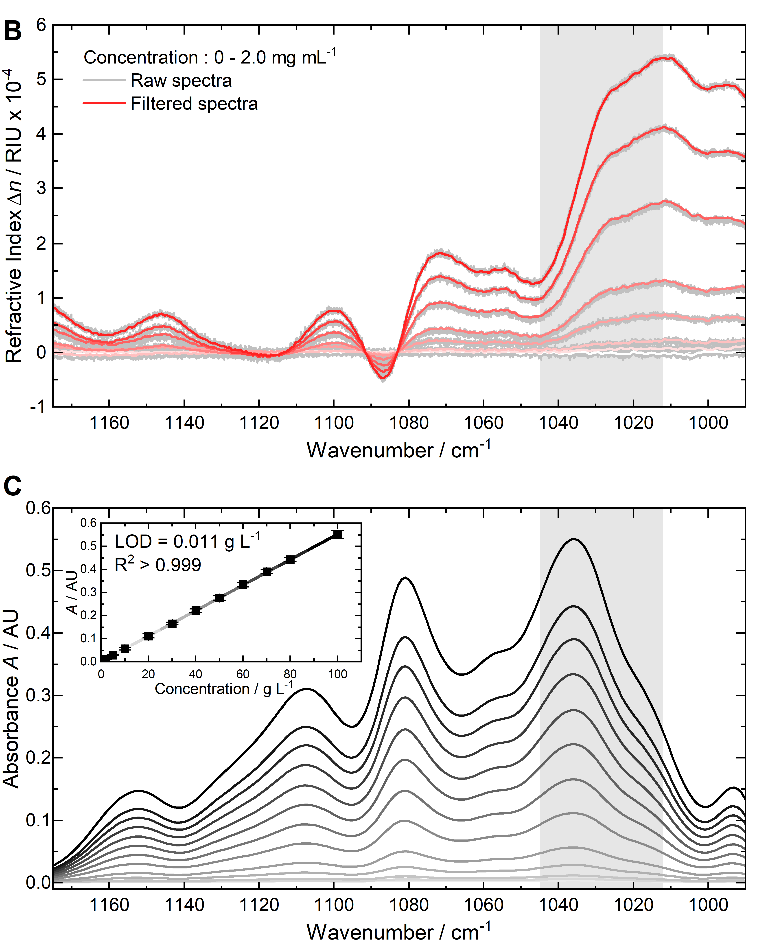


**Figure S2.** (A) IR dispersion spectra of glucose in aqueous solution (0.05 – 40 g L^-1^) recorded in transmission (d=170 µm) with the EC-QCL – MZI setup. Nine measurements were averaged per each calibration point. (B) A close-up view of the IR dispersion spectra recorded for the lowest concentrations of glucose, depicting the raw signal quality and the corresponding Savitzky-Golay filtered spectra, which were used for quantitative analysis (order: 3, window: 85 points / ~2 cm^-1^). (C) Reference FTIR absorbance spectra of glucose in aqueous solution (0.05 – 100 g L^-1^) recorded in transmission (d=25 µm) with Vertex 80v.


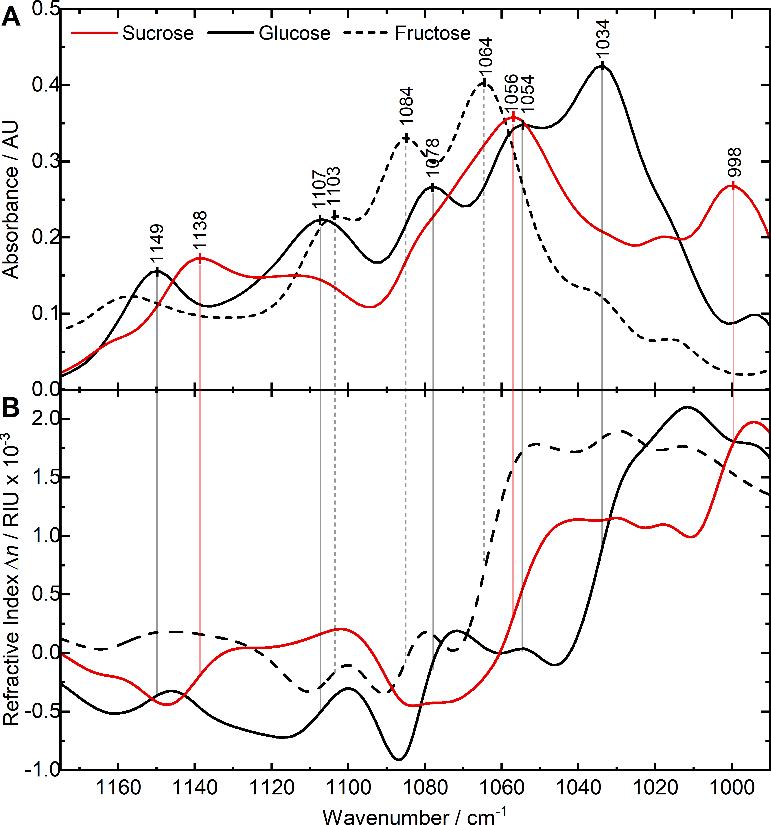


**Figure S3.** (A) Reference absorption spectra of 75 g L^-1^ sucrose, glucose, and fructose recorded by Vertex 80v in transmission (d=25 µm). (B) Dispersion spectra of 10 g L^-1^ sucrose, fructose, and glucose recorded by laser-based dispersion setup. Markers indicate the positions of characteristic absorption peaks and the corresponding regions of anomalous dispersion.


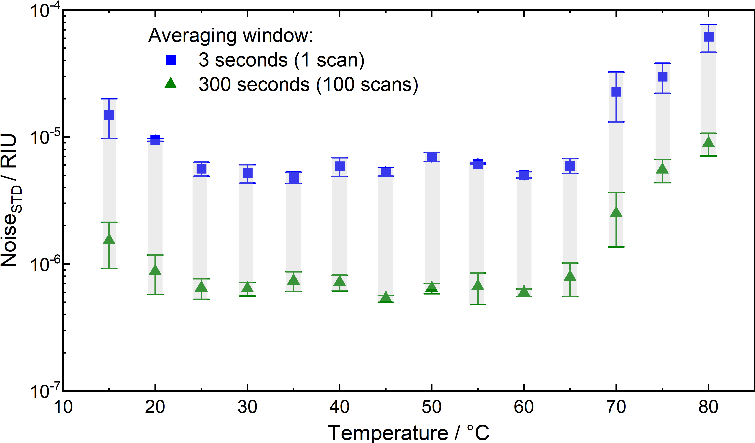


**Figure S4.** Impact of the liquid transmission cell’s temperature on the noise level of the dispersion spectrometer. The gray shaded areas indicate the range of noise values for averaging time ranging from 3 to 300 seconds at their respective temperatures.


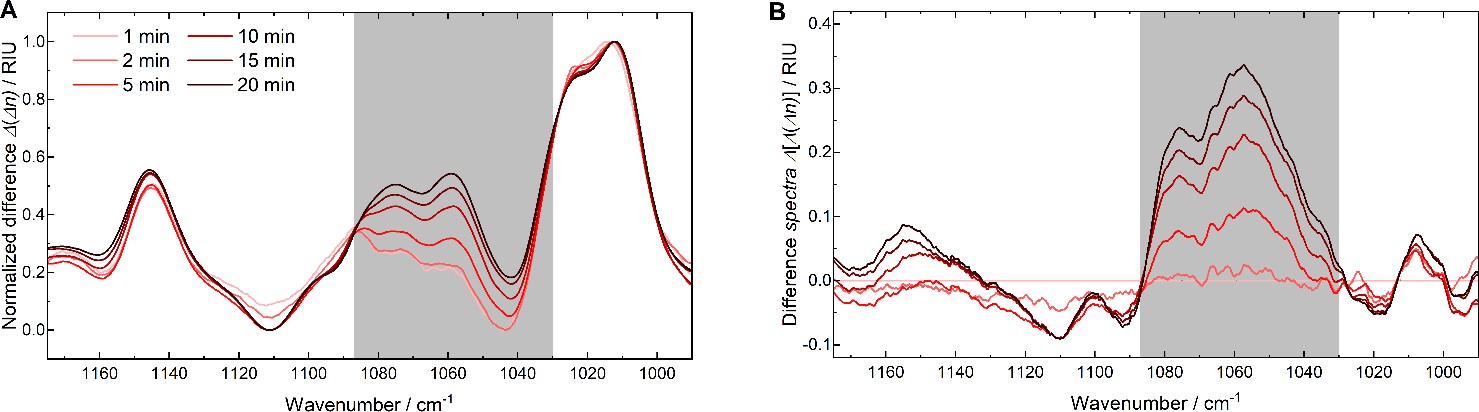


**Figure S5.** (A) Comparison of the normalized difference spectra of the enzymatic reaction catalyzed by invertase at selected reaction time stamps. Following the normalization process, spectral changes show significant inconsistency and variability across the highlighted spectral range, suggesting the presence of an additional component (reaction) taking place concurrently with the primary reaction. (B) Difference spectra calculated by subtracting the normalized difference spectrum at 1 minute from the difference spectra at 2-, 5-, 10-, 15-, and 20 minutes.


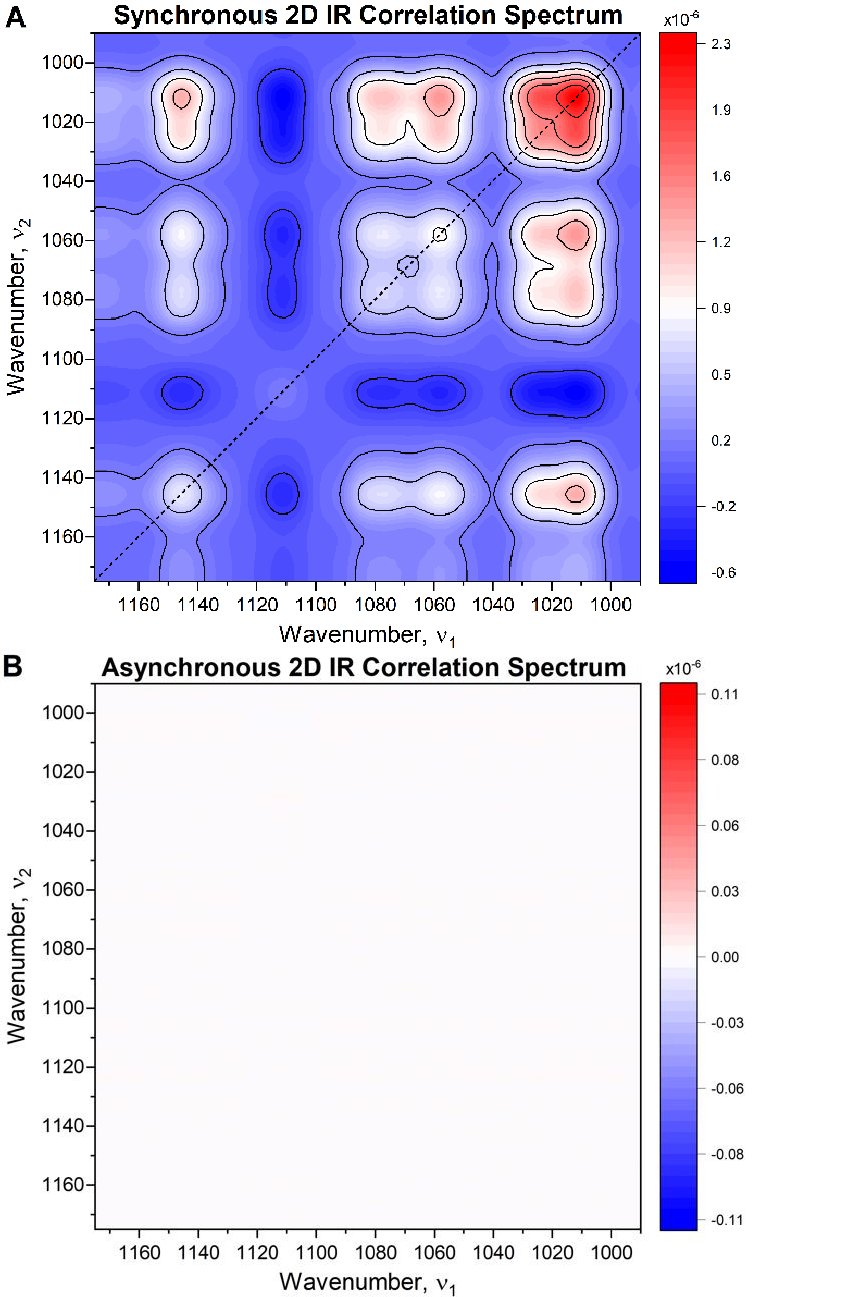


**Figure S6.** (A) Synchronous and (B) asynchronous 2D IR correlation maps calculated* from spectra of pure reaction components, assuming no mutarotation of the reaction products and hydrolysis of sucrose following reaction progression parameters derived in section 3.2 of the main manuscript. The peaks in the synchronous map coincide with those presented in the corresponding map for the enzymatic reaction (section 3.4). The absence of peaks in the asynchronous map serves as confirmation that all the visible peaks in the asynchronous map for the enzymatic reaction (section 3.4) arise from spectral changes related to mutarotation, a factor not considered in the calculations. The comparison between Fig. S6B and Fig. 5B was conducted using the same scale.

**Calculations were carried out as follows: In Step 1, spectra of 10 g L^-1^ α-D-glucose, and 10 g L^-1^ D-fructose were subtracted from a spectrum of 20 g L^-1^ sucrose, generating a difference spectrum of the reaction components. In Step 2, the resulting difference spectrum was multiplied by the reaction progression parameters, according to y=a[1-exp(-bt)] extracted from Fig. 3C. This step generated the time-dependent difference spectra for each reaction time stamp t from 0 to 20 min, which represented the initial conversion from sucrose to glucose and fructose without considering the subsequent mutarotation reactions. In Step 3, the calculated spectra were submitted to 2D-COS analysis.*
